# Molecular Characterization of Direct Target Genes and *cis*-Acting Consensus Recognized by Quorum-Sensing Regulator AphA in *Vibrio parahaemolyticus*

**DOI:** 10.1371/journal.pone.0044210

**Published:** 2012-09-12

**Authors:** Fengjun Sun, Yiquan Zhang, Li Wang, Xiaojuan Yan, Yafang Tan, Zhaobiao Guo, Jingfu Qiu, Ruifu Yang, Peiyuan Xia, Dongsheng Zhou

**Affiliations:** 1 Department of Pharmacy, Southwest Hospital, Third Military Medical University, Chongqing, People’s Republic of China; 2 State Key Laboratory of Pathogen and Biosecurity, Beijing Institute of Microbiology and Epidemiology, Beijing, People’s Republic of China; 3 School of Public Health, Chongqing Medical University, Chongqing, People’s Republic of China; Louisiana State University and A & M College, United States of America

## Abstract

**Background:**

AphA is the master quorum-sensing (QS) regulator operating at low cell density in vibrios. Molecular regulation of target genes by AphA has been characterized in *Vibrio harveyi* and *V. cholerae*, but it is still poorly understood in *V. parahaemolyticus*.

**Methodology/Principal Findings:**

The AphA proteins are extremely conserved in *V. parahaemolyticus, Vibrio* sp. Ex25, *Vibrio* sp. EJY3, *V. harveyi, V. vulnificus, V. splendidus, V. anguillarum, V. cholerae,* and *V. furnissii.* The above nine AphA orthologs appear to recognize conserved *cis*-acting DNA signals which can be represented by two consensus constructs, a 20 bp box sequence and a position frequency matrix. *V. parahaemolyticus* AphA represses the transcription of *ahpA, qrr4*, and *opaR* through direct AphA-target promoter DNA association, while it inhibits the *qrr2-3* transcription in an indirect manner. Translation and transcription starts, core promoter elements for sigma factor recognition, Shine-Dalgarno sequences for ribosome recognition, and AphA-binding sites (containing corresponding AphA box-like sequences) were determined for the three direct AphA targets *ahpA, qrr4,* and *opaR* in *V. parahaemolyticus*.

**Conclusions/Significance:**

AphA-mediated repression of *ahpA*, *qrr2-4*, and *opaR* was characterized in *V. parahaemolyticus* by using multiple biochemical and molecular experiments. The computational promoter analysis indicated the conserved mechanism of transcriptional regulation of QS regulator-encoding genes *ahpA*, *qrr4*, and *opaR* in vibrios.

## Introduction

Quorum sensing (QS) is a process of bacterial gene regulation in response to the fluctuations in cell-population density, by synthesizing, releasing, and detecting signal molecules called autoinducers that are induced with the increasing of cell density [1]. QS is employed by bacteria to regulate a diverse array of physiological activities including symbiosis, virulence, competence, conjugation, antibiotic production, motility, and biofilm formation [1].

**Figure 1:**
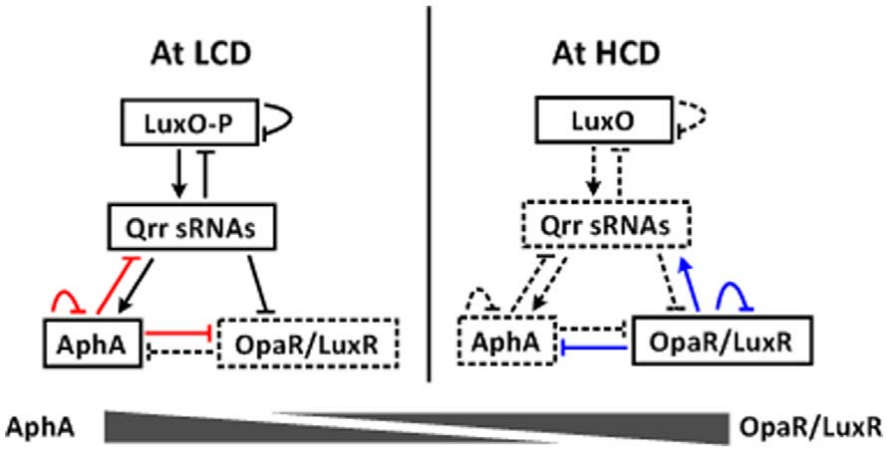
Model for signal transduction of QS systems in V. harveyi/V. parahaemolyticus. The details for signal transduction during QS have been described in text. At LCD, redundant Qrr sRNAs promote AphA translation and meanwhile inhibit LuxR translation. AphA further represses the transcription of luxR/opaR, qrr2-3, and its own gene. At HCD, the cessation of Qrr sRNA production leads to no production of AphA, and LuxR/OpaR translation occurs. LuxR/OpaR in turns represses the aphA transcription, and also feeds back to inhibit its own expression. Thus, AphA acts as a master regulator of QS behaviors at LCD, and in contrast, LuxR/OpaR is the major one operating at HCD; reciprocal gradients of AphA and LuxR/OpaR are established for controlling gene expression during transition between LCD and HCD. LuxR/OpaR is also able to activate the transcription of qrr2-4 genes, leading to rapid down-regulation of luxR/opaR [47]. It should be noted that this LuxR-qrr feedback dramatically accelerates the transition HCD to LCD, but it has no effect on QS behaviors at steady-state LCD or HCD [47].The dotted lines indicated the inhibited expression of relevant regulators or the cease of relevant regulatory cascades. The blue and red lines show the regulatory cascades that were experimentally validated in V. parahaemolyticus in our previous [15] and present works, respectively.

Vibrio harveyi, a pathogen of fishes and invertebrates, has been used as a model for QS studies. At low cell density (LCD) (Fig. 1), low concentrations of autoinducers lead to phosphorylation of LuxO (LuxO-P), and LuxO-P activates expression of the five qrr genes encoding sRNAs Qrr1-5 [2], [3]. The Qrr sRNAs promote the translation of AphA, while inhibit that of LuxR [4], [5], [6]. AphA further represses the luxR transcription, and moreover, overproduced AphA feeds back to inhibit the qrr transcription [7]. In addition, the over-production of Qrr sRNAs and LuxO-P triggers three additional feedback regulatory loops: i) LuxO-P represses the transcription of its own gene, ii) Qrr sRNAs inhibits the translation of luxO, and iii) Qrr sRNAs repress the translation of luxMN encoding the membrane-anchoring autoinducer-binding receptor protein LuxM and its cognate receptor LuxN [8], [9], [10]. The above feedbacks will contribute to control the Qrr levels within physiological states [10]. At high cell density (HCD) (Fig. 1), high concentrations of autoinducers reverse the phosphate flow in the circuit, leading to the dephosphorylation of LuxO. Dephosphorylated LuxO is inactive as a regulator, leading to the cessation of Qrr sRNA production. In the absence of Qrr sRNAs, AphA is not produced, but the translation of LuxR occurs [4], [5]. LuxR further represses the transcription of aphA, and moreover, overproduced LuxR feeds back to repress its own transcription [11]. The above regulatory circuits results in the reciprocal gradients of production of AphA and LuxR: maximal AphA and minimal LuxR production occur at LCD, while minimal AphA and maximal LuxR production at HCD [7]. Thus, AphA and LuxR represent the master QS regulators operating at LCD and HCD, respectively.

V. parahaemolyticus is a member of the Harveyi clade that is composed of eight characterized closely related Vibrio species including V. harveyi [12]. In recent years, V. parahaemolyticus has been recognized as the leading cause of infectious gastrointestinal illness via the fecal-oral route especially in coastal countries and regions [13]. Most infections are due to the eating of raw or undercooked seafood. Less commonly, infections in the skin occur when an open wound is exposed to warm seawater containing this pathogen. All the V. harveyi QS components can be annotated to be intact and highly conserved in the genome of V. parahaemolyticus [14]. Thus, the QS signal transduction cascades should be conserved in V. harveyi and V. parahaemolyticus [15] (see also Fig. 1).

The characterized orthologs of V. harveyi LuxR often have distinct names in different vibrios, and thus, the LuxR orthologs are referred to as HMRs (HCD master regulators) herein. The four HMRs, V. parahaemolyticus OpaR, V. harveyi LuxR, V. vulnificus SmcR, and V. alginolyticus ValR, are extremely conserved (>92% identity in amino acid sequences between each other), and they recognize a cis-acting consensus that can be annotated as a 20 bp invert-repeat box sequence TATTGATAAA-TTTATCAATA [15]. The OpaR box-like sequences can be found within the promoter-proximal DNA regions of opaR, qrr2-4 and aphA in V. parahaemolyticus, and the direct transcriptional regulation of these target genes by OpaR was further experimentally validated in V. parahaemolyticus [15]. As expected, the above characterized regulatory circuits are shared by V. harveyi and V. parahaemolyticus (Fig. 1).

To the best of our knowledge, there is no report on AphA in V. parahaemolyticus. The aphA (VP2762) gene [14] of V. parahaemolyticus is composed of an open reading frame containing 540 nucleotides with a G+C content of 44.4%, and it encodes a deduced protein of 180 amino acids (a.a.) with a calculated molecular mass of 20506.36 Da and with an isolectric point of 5.501. In the present work, two consensus constructs were built to represent conserved cis-acting signals recognized by AphA orthologs from nine Vibrio species including V. parahaemolyticus, and then AphA-mediated transcriptional regulation of QS regulator-encoding genes ahpA, qrr2-4, and opaR were characterized in V. parahaemolyticus.

## Materials and Methods

### Bacterial Strains

The wild-type *V. parahaemolyticus* strain RIMD 2210633 (WT) is a pandemic O3:K6 strain isolated from a patient with traveler’s diarrhea in Japan in 1996 [14]. The base pairs (bps) 2 to 516 of coding region (540 bp in total length) of *aphA* was deleted from WT to generate a nonpolar mutant strain *ΔaphA* (see also Fig. S1), using the suicide plasmid pDS132 [16] by homologous recombination as described previously [17], [18]. Briefly, the two DNA fragments (361 and 420 bp in length, respectively) flanking the 515 bp deletion region were amplified by PCR, purified, and used as the templates to create a 791 bp deletion construct that was subsequently inserted between *Pst*I and *Sph*I sites of pDS132. All the primers used in the present work were listed in Table 1. Upon being verified by DNA sequencing, the recombinant vector (containing deletion construct, and *sacB* gene conferring sensitivity to sucrose) was introduced into *Escherichia coli* S17-1(pir), and then transferred into WT by conjugation. The mutant strain was selected using resistance to 10% sucrose and sensitivity to 5 µg/ml chloramphenicol, and further verified by PCR.

**Table 1:** Oligonucleotide primers used in this study.

Target	Primers (forward/reverse, 5'-3')
**Construction of mutant**	
*aphA*	GTGACTGCAGCGCAGCAAATAACCAGAC/CCAATCACTTCAAGTTCTGTTGTCTTCAATCCAAATGGTC
*aphA*	GACCATTTGGATTGAAGACAACAGAACTTGAAGTGATTGG/GTGAGCATGCGTTTTCGTGACCGCTGTG
*aphA*	GTGACTGCAGCGCAGCAAATAACCAGAC/GTGAGCATGCGTTTTCGTGACCGCTGTG
**Complementation of mutant**	
*aphA*	AGCGGGATCCATGTCATTACCACACGTAATC/AGCGAAGCTTTTAACCAATCACTTCAAGTTC
**Protein expression**	
*aphA*	AGCGGGATCCATGTCATTACCACACGTAATC/AGCGAAGCTTTTAACCAATCACTTCAAGTTC
**EMSA**	
*aphA*	AACTTCCAACCACATAATTGCG/GGCTGGAGCAGGTATGATTG
*opaR*	TGTGGGTTGAGGTAGGTCG/GCCTAGTTCTAGGTCTCTTTGC
*qrr2*	AGTGGTTGCTTATGAATC/GGTCGAGAAGTATTATGC
*qrr3*	GGATAAGTTCAAATTGGATC/GTGGTTTCTGTGACATAC
*qrr4*	AACCGTGAAATCCATTTAC/CGACGCATTATTAACCAG
**DNase I footprinting**	
*aphA*	AACTTCCAACCACATAATTGCG/GGCTGGAGCAGGTATGATTG
*opaR*	AGTGGGTTGAAAGTCACATCC/GCCTAGTTCTAGGTCTCTTTGC
*qrr4*	AACCGTGAAATCCATTTAC/CGACGCATTATTAACCAG
**Primer extension**	
*aphA*	/GCTCTTACTGGCGCTTGAG
*opaR*	/ATCCATTTTCCTTGCCATTTG
*qrr2*	/TTATTGTGAACAATCTATAT
*qrr3*	/AATCAAGTTCACTAACAAC
*qrr4*	/ATATACTTGTGAACAATGTG
**LacZ fusion**	
*aphA*	GCGCGTCGACCATTCGTAATACAAAAGG/GCGCGGTACCTTCCAGAAGTAACCGATGCTAG
*opaR*	GCGCGTCGACTCCATCGTGTTGCCGTAGC/GCGCGGTACCCAATATCTGCGTGACCACCAC
*qrr2*	GCGCGTCGACAAAGTATGAAATAGTGTCGTAG/GCGCGAATTCTAGCCAACCGCAATAATC
*qrr3*	GCGCGTCGACGTGTTGATACCCATTATTC/GCGCGAATTCTGCGATTGGCTTATATAC
*qrr4*	GGGGTCGAC AACCGTGAAATCCATTTAC/GGGGAATTCATATACTTGTGAACAATGTG

A PCR-generated DNA fragment containing the *aphA* coding region together with its promoter-proximal DNA region (499 bp upstream of the coding sequence) and transcriptional terminator (285 bp downstream) were cloned between *Sal*I and *Sph*I sites of the pBRMob vector which is the ligation product of a 3219 bp fragment (containing the RP4 mob DNA region for plasmid mobilization) from pDS132 digested with *Hind*III, and the *Hind*III-digested plasmid pBR328 (harboring a chloramphenicol resistance gene) [19]. Upon being verified by DNA sequencing, the recombinant plasmid was introduced into *ΔaphA*, yielding the complemented mutant strain *C-aphA*.

### Bacterial Growth

For bacterial growth and maintenance, bacteria were cultivated in Luria-Bertani (LB) broth or on LB agar both with addition of 2% NaCl at 37°C. For longtime storage, bacteria were stored in Difco™ Marine (MR) broth 2216 (BD Bioscience) with addition of 30% glycerol at −85°C. Antibiotics were used at final concentrations of 100 µg/ml gentamicin, and 20 µg/ml chloramphenicol.

For the following biochemical assays, we employed a two-round design to prepare the liquid bacterial seed: firstly, the glyceric stock of bacterial cells was inoculated into 3 ml of MR broth for growing at 30°C with shaking at 200 rpm for 12 to 14 h to enter the stationary growth phase; secondly, the resulting cell culture was 50-fold diluted into 15 ml of fresh MR broth, and allowed to grow under the above conditions to reach an OD_600_ value of about 1.0 to 1.2. The seed culture was then 1,000-fold diluted into 30 ml of fresh MR broth for further growth under the above conditions. To determine the bacterial growth curves, the OD_600_ values were monitored for each culture with an 1 h interval. For the primer extension or LacZ fusion experiments, bacterial cells were harvested at an OD_600_ value of 0.1 to 0.15 to simulate LCD conditions.

**Figure 2:**
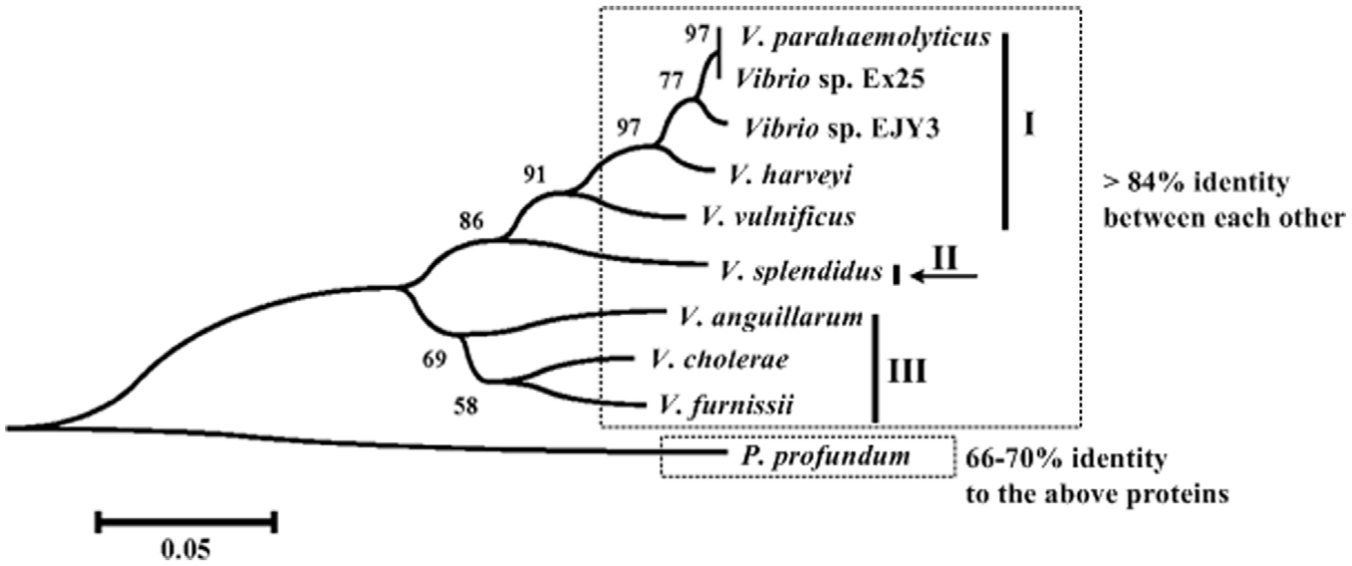
Phylogenetic tree of AphA orthologs. The protein sequences were derived from V. parahaemolyticus RIMD 2210633 [14], Vibrio sp. Ex25 (Accession numbers CP001805.1, and CP001806.1), Vibrio sp. EJY3 [48], V. harveyi ATCC BAA-1116 [49], V. vulnificus YJ016 [50], V. splendidus LGP32 [51], V. anguillarum 775 [52], V. cholerae N16961 [53], V. furnissii NCTC 11218 [54], and P. profundum SS9 [25]. The a.a. sequences were aligned by the CLUSTALW [55] web server at http://align.genome.jp/. The aligned sequences were then used to construct an unrooted neighbor-joining tree using MEGA version 5.0 [56] with a bootstrap iteration number of 1000. Shown on branch points of phylogenic tree were bootstrap values (%).

### LacZ Fusion and β-galactosidase Assay

The 300 to 800 bp promoter-proximal DNA region of each indicated gene was amplified by PCR with ExTaq™ DNA polymerase (Takara) using RIMD 2210633 genome DNA as the template. PCR fragments were then directionally cloned between *Sal*I and *EcoR*I (or *Kpn*I) sites of low-copy-number plasmid pHRP309 that harbors a gentamicin resistance gene and a promoterless *lacZ* reporter gene [20]. Correct cloning was verified by DNA sequencing. An empty pHRP309 plasmid was also introduced into each strain tested as the negative control. The *V. parahaemolyticus* strains transformed with recombinant plasmids or empty pHRP309 were grown as above to measure the β-galactosidase activity in cellular extracts using a β-Galactosidase Enzyme Assay System (Promega) [21].

### RNA Isolation and Primer Extension Assay

Total bacterial RNAs were extracted using the TRIzol Reagent (Invitrogen) [15], [21]. RNA quality was monitored by agarose gel electrophoresis, and RNA quantity was determined by spectrophotometry.

**Table 2:** Known or predicted direct targets of AphA.

Gene name	Gene ID	AphA box-like sequence	Position^&^	Score	Reference	Regulation by AphA
***V. parahaemolyticus*** ** RIMD 2210633**						
*aphA*	VP2762	GTATTCCACTTCATGCTTAT	D-238…-219	16.37	This study	Negative
*opaR*	VP2516	ATATGCACCATTACACTCAT	D-98…-79	18.32	This study	Negative
*qrr4*	VPA0199-0200 intergenic	ATATGCCACTTGTTACATTG	R-146…-127	10.96	This study	Negative
***Vibrio*** ** sp. Ex25**						
*aphA*	VEA_002309	CTATTCCACTTCATGCTTAT	D-240…-221	15.71	Predicted	Negative
*luxR*	VEA_002553	ATATGCACCATTACACTCAT	D-161…-142	18.32	Predicted	Negative
*qrr4*	VEA000800-000799 intergenic	ATATGCCACTTCATGCACTG	R-146…-127	14.05	Predicted	Negative
***Vibrio*** ** sp. EJY3**						
*aphA*	VEJY3_14120	GTATTCCACTTCATGCTTAT	D-242…-223	16.37	Predicted	Negative
*luxR*	VEJY3_12990	ATATGCACCATTACACTCAT	D-98…-79	18.32	Predicted	Negative
*qrr4*	VEJY316416-16421 intergenic	ATATGCTACCAACCGCATTG	R-145…-126	7.64	Predicted	Negative
***V. harveyi*** ** ATCC BAA-1116**						
*aphA*	VIBHAR_00046	CTATTCCACTTCATGCTTAT	D-240…-221	15.71	[7]	Negative
*luxR*	VIBHAR_03459	ATATGCACCATTACACTCAT	D-101…-82	18.32	[7]	Negative
*qrr4*	VIBHAR_06697	ATATGCCACCATGCGCATTG	R-146…-127	11.01	[7]	Negative
***V. vulnificus*** ** YJ016**						
*aphA*	VV3005	CTATTCCACTTTATGCTTAT	D-238…-219	16.45	Predicted	Negative
*smcR*	VV2770	ATATGCACCATTACACTCAT	D-110…-91	18.32	Predicted	Negative
*qrr4*	VVA0457-0458 intergenic	ATACACACAAATTTGCATAT	R-97…-78	8.65	Predicted	Negative
***V. splendidus*** ** LGP32**						
*aphA*	VS_2891	GTATTCTACTTTATGCTTAT	D-237…-218	15.94	Predicted	Negative
*luxR*	VS_2539	ATATGCACCATTACACTCAT	D-79…-60	18.32	Predicted	Negative
***V. anguillarum*** ** 775**						
*aphA*	VAA_02615	GTATTCTACTTTATGCTTAT	D-253…-234	15.94	Predicted	Negative
*vanT*	VAA_00743	ATATGCAGCAGTACACTCAT	D-99…-80	13.81	Predicted	Negative
***V. cholerae*** ** N16961**						
*aphA*	VC2647	GTATTCCACTTTATGCTTAT	D-245…-226	17.11	[30]	Negative
*hapR*	VC0583	ATATGCACCATTACACTCAT	D-101…-82	18.32	Predicted	Negative
*pva*	VCA0877	ATATGCAACAAATTACACAT	D-178…-159	12.75	[44]	Negative
*alsR*	VC1588	ATATACACACAGATTCATAT	R-93…-74	8.6	[29]	Negative
*alsD*	VC1589	ATATACACACAGATTCATAT	D-32…-13	8.6	[29]	Negative
*vpsT*	VCA0952	ATACGCAAAAAGACTCTTAT	R-242…-223	10.7	[28]	Positive
*tcpP*	VC0826	TTATGCAATTAAGTTCTCAT	D-110…-91	6.71	[27]	
***V. furnissii*** ** NCTC 11218**						
*aphA*	vfu_A00617	GTATTCTACTTTATGCTTAT	D-246…-227	15.94	Predicted	Negative
*luxR*	vfu_A00883	ATGTACGCAATTACACTCAT	D-104…-85	9.66	Predicted	Negative

&, ‘D’ indicates the direct sequence, and the minus numbers denote nucleotide positions upstream of genes.

**Figure 3:**
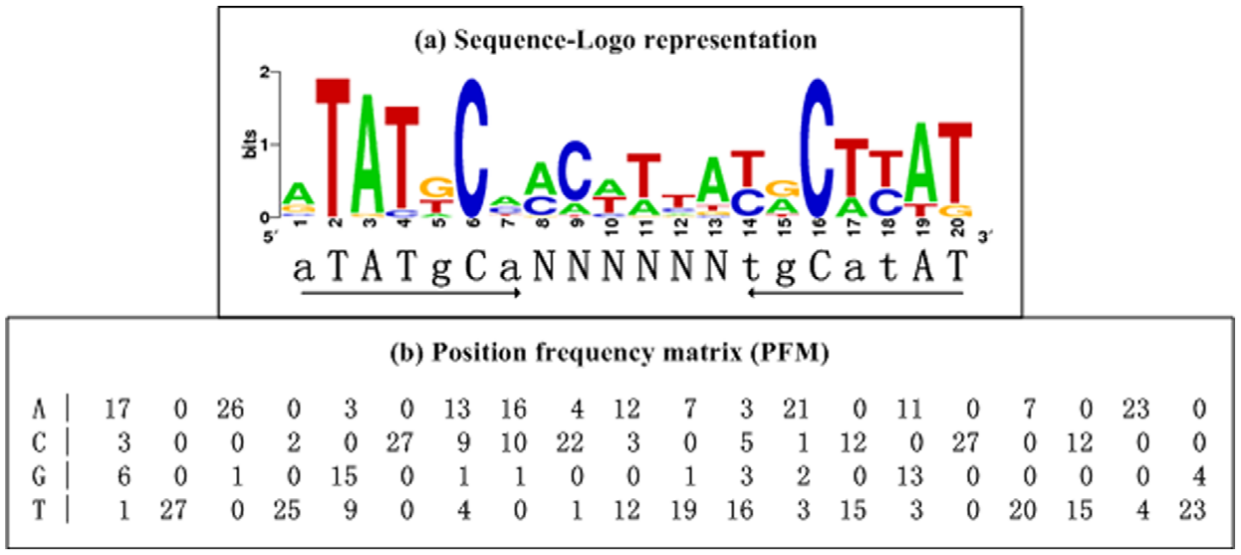
Cis-acting consensus constructs. (a) The sequence logo representation of AphA sites (Table 2) was generated by WebLogo [24]. The 20-bp box sequence ATATGCAN_6_ TGCATAT contained imperfect inverted repeats of ATATGCA with a 6-nt centered spacer. (b) A position frequency matrix describes the alignment of AphA sites, and denotes the frequency of each nucleotide at each position.

For the primer extension assay [15], [21], an oligonucleotide primer complementary to a portion of RNA transcript of each indicated gene was employed to synthesize cDNAs from the RNA templates. Ten to 100 µg of total RNA from each strain was annealed with 1 pmol of [γ-^32^P] end-labeled reverse primer using a Primer Extension System (Promega) according to the manufacturer’s instructions. The same labeled primer was also used for sequencing with a fmol® DNA Cycle Sequencing System (Promega). The primer extension products and sequencing materials were concentrated and analyzed in a 6% polyacrylamide/8 M urea gel. The result was detected by autoradiography (Kodak film).

**Figure 4:**
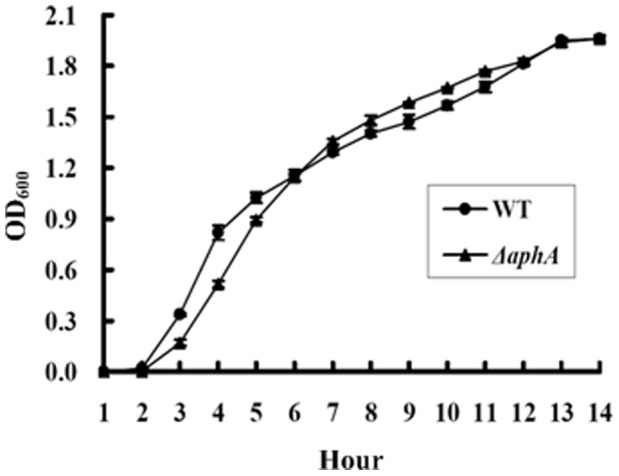
Bacterial growth curves. A two-round design to prepare the bacterial seed culture was employed: firstly, the glyceric stock of bacterial cells was inoculated into 3 ml of MR broth for growing at 30°C with shaking at 200 rpm for 12 to 14 h to enter the stationary growth phase; secondly, the resulting cell culture was 50-fold diluted into 15 ml of fresh MR broth, and allowed to grow under the above conditions to reach an OD_600_ value of about 1.0 to 1.2. The seed culture was then 1000-fold diluted into 30 ml of fresh MR broth for the further growth under the above conditions, and the OD_600_ values were monitored for each culture with an 1 h interval.

### Preparation of Purified AphA Protein

The preparation of purified AphA protein was done as described previously [7] with the omission of thrombin cleavage procedures. The entire coding region of *aphA* of strain RIMD 2210633 was cloned between *BamH*I and *Hind*III sites of plasmid pET28a (Novagen). The recombinant plasmid encoding 6× His-tagged AphA protein (His-AphA) was transformed into *E. coli* BL21λDE3 cells, and grown in LB broth at 37°C with shaking at 200 rpm for 4 to 5 h. The resulting culture was diluted 1/100 into 200 to 300 ml of fresh LB broth, and grown under the above conditions to an OD_600_ of about 0.5. The culture was shifted to 18°C for 1 h, and then induced with 1 mM IPTG for 16 to 18 h with shaking at 100 rpm. Cells were collected by centrifugation and frozen at −60°C. The pellet was resuspended in 10 ml of 50 mM sodium phosphate buffer, pH 7.4, 500 mM NaCl, and 5 mM imidazole. Cells were disrupted using a cell cracker, and the insoluble material was pelleted by centrifugation at 15,000 rpm. The clarified supernatant was applied to a 3 ml Ni-NTA Agarose Column (Qiagen), and the overproduced protein was purified under native conditions. Fractions from a homogenous peak were pooled, and the final preparation was dialyzed against 10 mM Tris HCl, pH 7.5, 10 mM NaCl, 1 mM EDTA, 0.1 mM DTT, and 20% glycerol. The purified protein was stored at −60°C, and the protein purity was verified by SDS-PAGE.

### Gel Mobility Shift Assay (EMSA)

The 300 to 600 bp promoter-proximal DNA region of each indicated gene was amplified by PCR. For EMSA [15], [21], the 5′ ends of DNA were labeled using [γ-^32^P] ATP and T4 polynucleotide kinase. DNA binding was performed in a 10 µl reaction volume containing binding buffer [1 mM MgCl_2_, 0.5 mM EDTA, 0.5 mM DTT, 50 mM NaCl, 10 mM Tris-HCl (pH 7.5) and 0.05 mg/ml poly-(dI-dC)], labeled DNA (1000 to 2000 c.p.m/µl), and increasing amounts of His-AphA. Three controls were included in each EMSA experiment: 1) cold probe as specific DNA competitor (the same promoter-proximal DNA region unlabeled), 2) negative probe as non-specific DNA competitor (the unlabeled coding region of the 16S rRNA gene), and 3) non-specific protein competitor [rabbit anti-F1-protein polyclonal antibodies]. The F1 protein is the protective antigen from *Yersinia pestis* [22]. After incubation at room temperature for 30 min, the products were loaded onto a native 4% (w/v) polyacrylamide gel, and electrophoresed in 0.5× TBE buffer for about 50 min at 220 V. Radioactive species were detected by autoradiography after exposure to Kodak film at −70°C.

### DNase I Footprinting

For DNase I footprinting [15], [21], the 250 to 600 bp promoter-proximal DNA regions with a single ^32^P-labeled end were PCR amplified with either sense or antisense primer being end-labeled. The PCR products were purified using the QiaQuick columns (Qiagen). Increasing amounts of His-AphA were incubated with the purified, labeled DNA fragment (2 to 5 pmol) for 30 min at room temperature, in a final 10 µl reaction volume containing the binding buffer used in EMSA. Before DNA digestion, 10 µl of Ca^2+^/Mg^2+^ solution (5 mM CaCl_2_ and 10 mM MgCl_2_) was added, followed by incubation for 1 min at room temperature. The optimized RQ1 RNase-Free DNase I (Promega) was then added to the reaction mixture, and the mixture was incubated at room temperature for 40 to 90 s. The reaction was quenched by adding 9 µl of stop solution (200 mM NaCl, 30 mM EDTA, and 1% SDS), followed by incubation for 1 min at room temperature. The partially digested DNA samples were extracted with phenol/chloroform, precipitated with ethanol, and analyzed in 6% polyacrylamide/8 M urea gel. Protected regions were identified by comparison with the sequence ladders. For sequencing, we used the *fmol*® DNA Cycle Sequencing System (Promega). The templates for sequencing were the same as the DNA fragments for DNase I footprinting assays. Radioactive species were detected as above.

**Figure 5:**
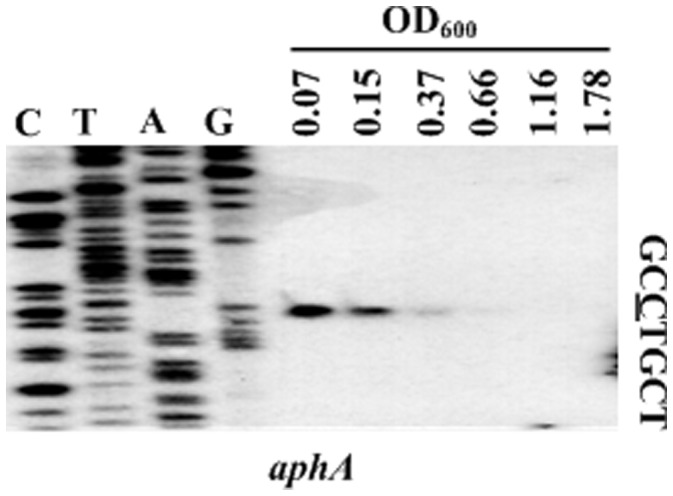
Transcriptional pattern of aphA during growth. The bacterial cells were harvested at various OD_600_ values during growth (according to the bacterial growth curves in Fig. 4) for total RNA isolation. An oligonucleotide primer was designed to be complementary to the RNA transcript of aphA. The primer extension products were analyzed with 8 M urea-6% acrylamide sequencing gel. Lanes C, T, A, and G represented Sanger sequencing reactions. The transcription start site of aphA was underlined in the DNA sequence.

### Computational Promoter Analysis

The 300 bp promoter region upstream of start codon of each indicated gene were retrieved with the ‘*retrieve-seq*’ program [23]. Known or predicted binding sites of AphA were collected and aligned to generate the position frequency matrix (PFM) by using the ‘*matrices-consensus*’ tool [23].The sequence logo representation of the above binding sites was generated by the WebLogo tool [24]. The ‘*matrices-paster*’ tool [23] was used to match the PFM within the promoter-proximal DNA regions tested.

### Experimental Replicates and Statistical Methods

For the growth curve and LacZ fusion assays, experiments were performed with at least three independent bacterial cultures, and values were expressed as mean ± standard deviation (SD). Statistical testing of difference was made by the Student's paired *t* test, and a *P* value of <0.01 was taken as significant. For the primer extension, EMSA, and footprinting assays, representative data from at least two independent biological replicates were shown.

**Figure 6:**
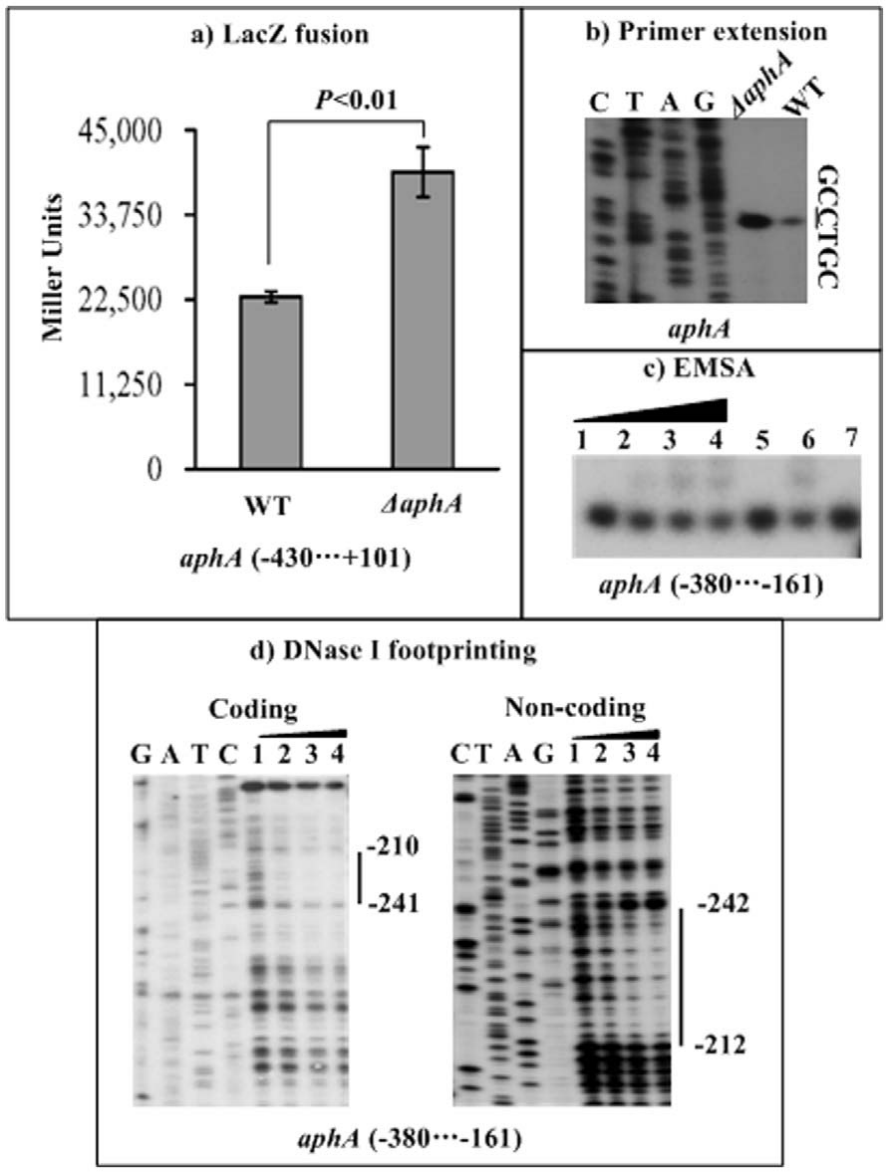
Repression of its own gene by AphA. a) LacZ fusion. The promoter-proximal DNA region of aphA was cloned into the lacZ transcriptional fusion vector pHRP309, and then transformed into WT or ΔaphA to determine the β-galactosidase activity in cellular extracts. Shown was the aphA promoter activity (Miller units) in ΔaphA or WT. **b) Primer extension.** An oligonucleotide primer was designed to be complementary to the RNA transcript of aphA. The primer extension products were analyzed with 8 M urea-6% acrylamide sequencing gel. Lanes C, T, A, and G represented Sanger sequencing reactions. The transcription start site of aphA was underlined in the DNA sequence. **c) EMSA.** The radioactively labeled DNA fragment from the 380th to 161st bp upstream of aphA was incubated with increasing amounts (lanes 1 to 4∶0, 15, 20, and 25 pmol, respectively) of purified His-AphA protein, and then subjected to 4% (w/v) polyacrylamide gel electrophoresis; the band of free DNA become weak with increasing amounts of His-AphA protein, and a retarded DNA band with decreased mobility turned up, which presumably represented the DNA-AphA complex. For lane 5, 2 pmol of cold probe, 25 pmol of His-AphA, and labeled target DNA fragment were added; the retarded DNA band disappeared due to the action of cold probe as specific DNA competitor. For lane 6, 2 pmol of negative probe, 25 pmol of His-AphA, and labeled target DNA fragment were added; the retarded DNA band occurred since the negative probe had no effect on the DNA-AphA complex. For lane 7, 20 pmol of non-specific protein competitor, and labeled target DNA fragment were added; there was no retarded DNA band observed. **d) DNase I footprinting.** Labeled coding or non-coding DNA probes were incubated with increasing amounts of purified His-AphA (Lanes 1, 2, 3, and 4 containing 0, 15, 20, and 25 pmol, respectively), and subjected to DNase I footprinting assay. Lanes G, A, T, and C represented Sanger sequencing reactions. The footprint regions were indicated with vertical bars. The negative numbers indicated nucleotide positions upstream of aphA.

**Figure 7:**
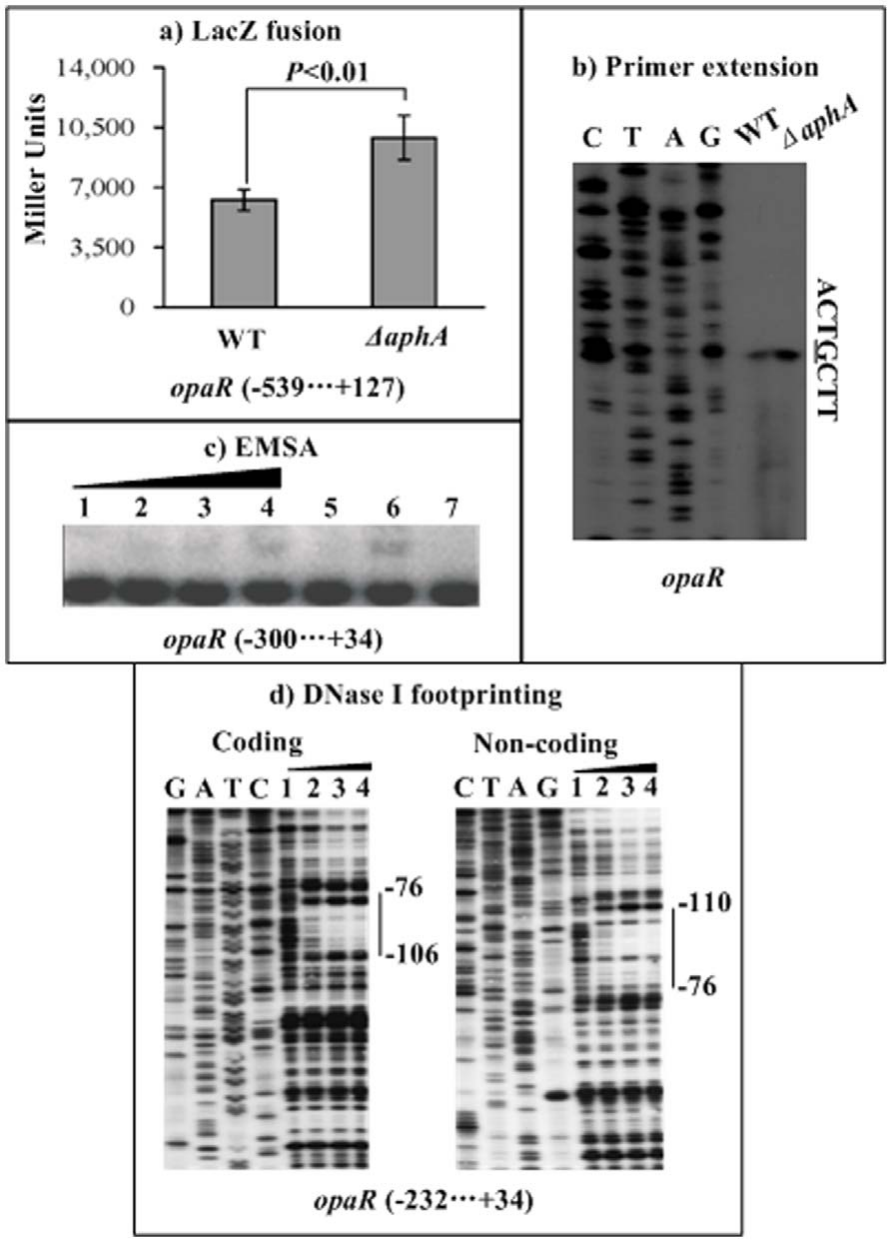
Repression of qrr2-4 by OpaR. For LacZ fusion (a1, b1, and c1), the promoter-proximal fragment of each of qrr2-4 was cloned into pHRP309, and then transformed into WT or ΔaphA to determine the β-galactosidase activity (Miller units) in cellular extracts. For primer extension (a2, b2, and c2), an oligonucleotide primer was designed to be complementary to the RNA transcript of each of qrr2-4. For EMSA (a3, b3, and c3) and DNase I footprinting (c4), the promoter-proximal fragment of each of qrr2-4 were radioactively labeled, and then incubated with increasing amounts of purified His-AphA protein. The experiments were done as described in Fig 6. The transcription start sites of qrr2-4 were underlined in the DNA sequence. Lanes G, A, T, and C represented Sanger sequencing reactions. The footprint regions were indicated with vertical bars. The negative or positive numbers indicated nucleotide positions upstream or downstream of relevant qrr gene, respectively.

## Results

### Cis-acting Consensus Recognized by AphA

The AphA orthologs from nine Vibrio species (V. parahaemolyticus, Vibrio sp. Ex25, Vibrio sp. EJY3, V. harveyi, V. vulnificus, V. splendidus, V. anguillarum, V. cholerae, and V. furnissii) with determined whole genome sequences gave high identity (≥84%) among each other in the a.a. sequences. A phylogenetic tree (Fig. 2) was constructed from the aligned a.a. sequences of the above nine orthologous AphA proteins, with an additional AphA homologue from Photobacterium profundum SS9 [25] as the outgroup (this protein has about 66 to 70% identity to the above nine AphA orthologs). Three clades I, II, and III were arbitrarily identified for the nine Vibrio species on the basis of phylogenetic tree.

Since the nine AphA orthologs are highly conserved, when they act as the DNA-binding regulatory proteins, they will recognize conserved cis-acting DNA signals in the relevant vibrios. Known or predicted binding sites of the nine AphA orthologs were collected (Table 2), and then aligned to generate an AphA consensus that manifested as a PFM (in which each row and column represents a position and a nucleotide, respectively) and as a 20 bp box sequence ATATGCAN_6_ TGCATAT that contained imperfect inverted repeats of ATATGCA with a 6-nt centered spacer (Fig. 3).

**Figure 8:**
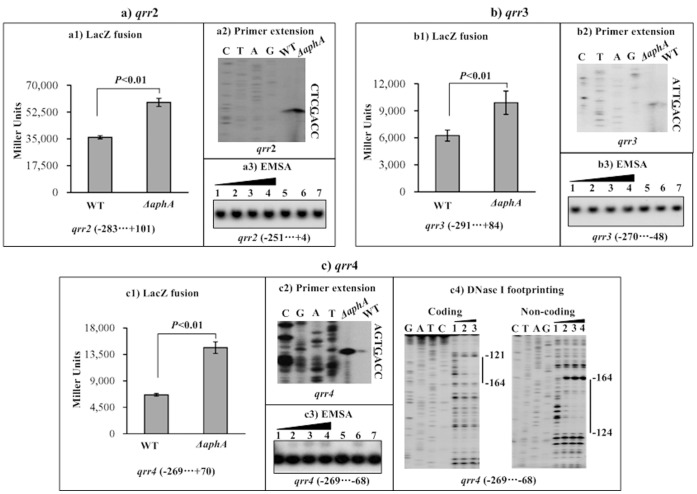
Repression of opaR by AphA. For LacZ fusion (a), the promoter-proximal DNA fragment of opaR was cloned into pHRP309, and then transformed into WT or ΔaphA to determine the β-galactosidase activity (Miller units) in cellular extracts. For primer extension (b), an oligonucleotide primer was designed to be complementary to the RNA transcript of opaR. For EMSA (c) and DNase I footprinting (d), the promoter-proximal DNA fragment of opaR was incubated with increasing amounts of purified His-AphA protein. The experiments were done as described in Fig 6. The transcription start site of opaR was underlined in the DNA sequence. Lanes G, A, T, and C represented Sanger sequencing reactions. The footprint regions were indicated with vertical bars. The negative or positive numbers indicated nucleotide positions upstream or downstream of opaR, respectively.

The presence of AphA box-like sequences within the promoter-proximal DNA regions of aphA, qrr4, and opaR in V. parahaemolyticus (Table 2) indicated that these QS regulators-encoding genes might be the direct AphA targets in V. parahaemolyticus, which were further validated by the following gene regulation experiments.

### Growth of WT and *ΔahpA*

The growth curves of WT and *ΔaphA* grown at 30°C in MR broth were determined (Fig. 4). *ΔaphA* grew slightly slower than WT from time of inoculation to mid-exponential growth phase; however, from mid-exponential phase to enter stationary phase, the two strains showed almost indistinguishable growth rates. These results indicated that the *aphA* mutation had little effect on bacterial *in vitro* growth when MR broth was used for cultivation.

**Figure 9:**
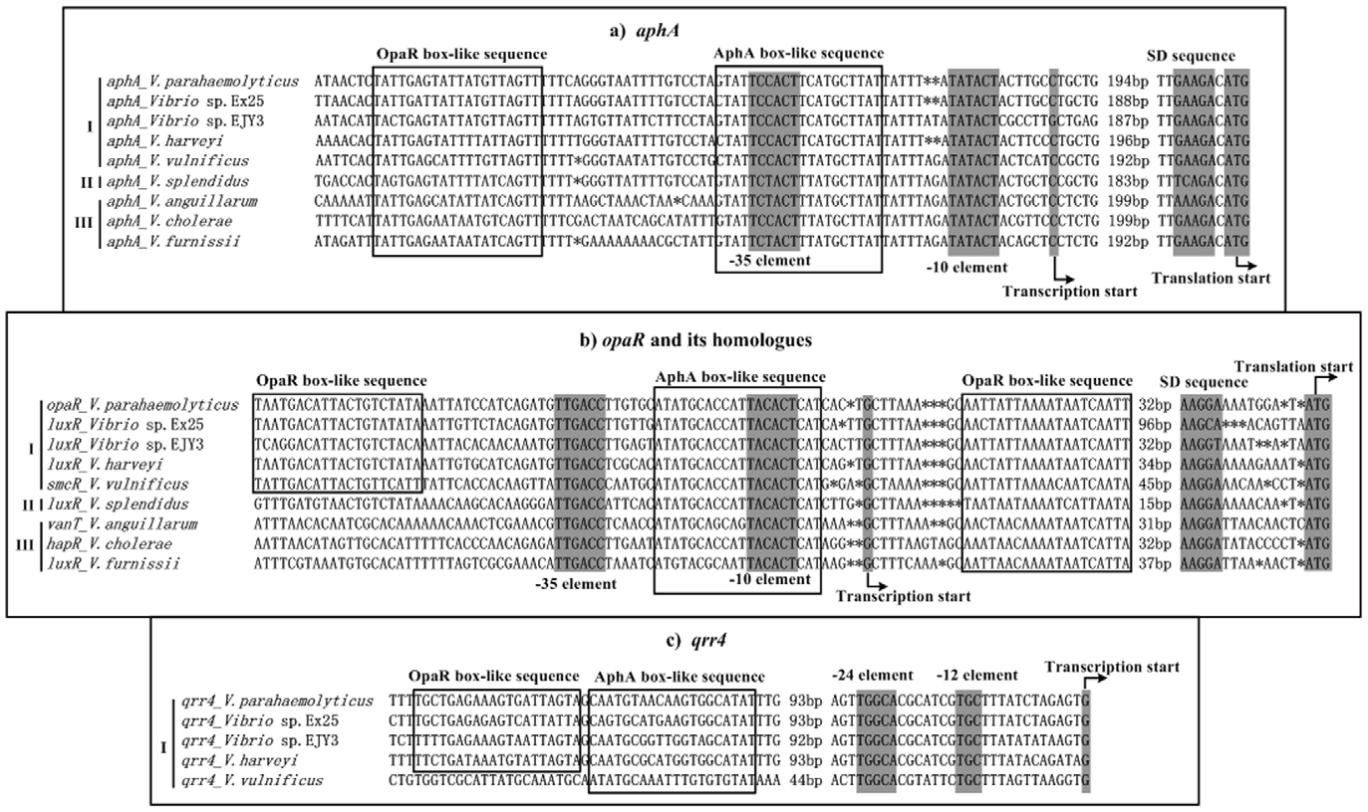
Organization of promoter-proximal DNA regions. DNA sequences were derived from V. parahaemolyticus RIMD 2210633 [14], Vibrio sp. Ex25 (Accession numbers CP001805.1, and CP001806.1), Vibrio sp. EJY3 [48], V. harveyi ATCC BAA-1116 [49], V. vulnificus YJ016 [50], V. splendidus LGP32 [51], V. anguillarum 775 [52], V. cholerae N16961 [53], and V. furnissii NCTC 11218 [54]. Shown were translation and transcription starts, SD sequences, AphA or OpaR box-like sequences, and –0/−12 and –35/−24 core promoter elements. The prediction of OpaR box-like sequences were described previously [15].

### Cell Density-dependent Expression of *aphA*

The mRNA levels of *aphA* were measured in WT grown at various OD_600_ values (i.e. at different cell densities) by the primer extension assay (Fig. 5). The primer extension experiments detected a single transcription start site located at 200 bp upstream of *aphA.* Therefore, a single promoter was transcribed for *aphA* under the growth conditions tested. The *aphA* mRNA levels decreased dramatically with the increasing of cell density, and almost no *aphA* mRNA could be detected at HCD (OD_600_≥1.16), which supported the model (as described Fig. 1) of cell density-dependent regulation of *aphA*.

For the following gene regulation experiments, bacteria cells were harvested at an OD_600_ value of 0.1 to 0.15 to simulate LCD conditions at which *aphA* was abundantly transcribed.

### Negative Auto-regulation of AphA

An *aphA::lacZ* fusion vector, containing a 531 bp promoter-proximal region of *aphA* and the promoterless *lacZ*, was transformed into WT or *ΔaphA* to compare the *aphA* promoter activities in these two strains (Fig. 6a). The LacZ fusion experiments disclosed that the *aphA* promoter activity significantly enhanced in *ΔaphA* relative to WT. The primer extension experiments (Fig. 6b) further disclosed that the mRNA level of *aphA* enhanced in *ΔaphA* relative to WT. A 220 bp promoter-proximal DNA region of *aphA* was subjected to EMSA with purified His-AphA protein (Fig. 6c). The results showed that His-AphA was able to bind to the target DNA fragment in a dose-dependent manner *in vitro*. As further determined by DNase I footprinting (Fig. 6d), His-AphA protected a single region from 242 to 210 bp upstream of *aphA* against DNase I digestion in a dose-dependent manner. This footprint contained a predicted AphA box-like sequence, and was considered as the AphA-binding site for *aphA*. Taken together, the AphA regulator was able to recognize the promoter of its own gene to directly repress its activity in *V. parahaemolyticus*.

### Repression *of opaR* by AphA

An *opaR::lacZ* fusion vector, containing a 666 bp promoter-proximal region of *opaR* and the promoterless *lacZ*, was transformed into both WT and *ΔaphA* to compare the *opaR* promoter activities in these two strains (Fig. 7a). The LacZ fusion experiments disclosed that the promoter activity of *opaR* significantly enhanced in *ΔaphA* relative to WT. The primer extension experiments (Fig. 7b) detected a single transcription start site located at 74 bp upstream of *opaR*, and further disclosed that the mRNA level of *opaR* enhanced in *ΔaphA* relative to WT. A 334 bp promoter-proximal DNA region of *opaR* was subjected to EMSA with purified His-AphA protein (Fig. 7c). The results showed that His-AphA was able to bind to the DNA fragment in a dose-dependent manner *in vitro*. As further determined by DNase I footprinting (Fig. 7d), His-AphA protected a single region from 110 to 76 bp upstream of *opaR* against DNase I digestion in a dose-dependent manner. This footprint contained a predicted AphA box-like sequence, and was considered as the AphA-binding site for *opaR*. Taken together, AphA was able to recognize the promoter of *opaR* to directly repress its activity in *V. parahaemolyticus*.

### Repression of *qrr2-4* by AphA

The LacZ fusion vector for each of *qrr2-4* was transformed into WT or *ΔaphA*, respectively, to compare the promoter activities of each *qrr* gene in the two strains (Fig. 8a1, 8b1, and 8c1). The results showed that the promoter activity of each of *qrr2-4* significantly enhanced in *ΔaphA* relative to WT. The primer extension experiments were then conducted to compare the yields of primer extension product of each of *qrr2-4* in WT and *ΔaphA* (Fig. 8a2, 8b2, and 8c2, respectively). The primer extension assay defined the transcription start sites for all of *qrr2*-*4*, and this assay also indicated that the mRNA level of each of *qrr2-4* enhanced in *ΔaphA* relative to WT. Each of the promoter-proximal DNA regions of *qrr2-4* was subjected to EMSA with the purified His-AphA protein (Fig. 8a3, 8b3, and 8c3, respectively). The results showed that His-AphA was able to bind to the DNA fragment of *qrr4* rather than *qrr2-3*. As further determined by DNase I footprinting (Fig. 8c4), His-AphA protected a single region from 164 to 121 bp upstream of *qrr4* against DNase I digestion in a dose-dependent manner. This footprint contained a predicted AphA box-like sequence, and was considered as the AphA-binding site for *qrr4*. Taken together, AphA was able to recognize the promoters of *qrr4* to directly repress its activity in *V. parahaemolyticus*; in contrast, the *qrr2-3* genes appeared to be repressed by AphA at the transcriptional level in an indirect manner.

## Discussion

### Master QS Regulators at LCD and HCD

In V. harveyi and V. cholerae (the causative agent of the frequently fatal epidemic diarrheal disease cholera), AphA acts as a LCD master regulator of QS behaviors, and in contrast, LuxR/HapR is the major one operating at HCD [7]. The present work also confirmed the maximum aphA transcription at LCD and the minimum one at HCD. It seems that the reciprocal, cell density-dependent expression of AhpA and HMR is a conserve mechanism used by vibrios during the QS signal transduction.

Control of biofilm formation and virulence by HMRs and AphA has been characterized in multiple vibrios. AphA is an activator of virulence and biofilm formation in V. cholerae [26], [27], [28], [29] and V. parahaemolyticus (data unpublished). The four HMRs, HapR, OpaR, LuxR, and SmcR, are the repressors of virulence in V. cholerae [30], [31], [32], [33], V. parahaemolyticus [34], [35], V. harveyi [36], and V. vulnificus [37], respectively. HapR and OpaR also repress biofilm formation in V. cholerae [38], [39], [40] and in the pandemic O3:K6 V. parahaemolyticus (data unpublished), respectively. It should be noted that the in vivo biofilm formation has been shown to contribute to the infectivity of V. cholerae upon oral ingestion [41], [42]. Taken together, the reciprocal, cell density-dependent expression of AphA and HMR are thought to be established for controlling the virulence-related gene expression during infection: on the initial colonization (i.e., LCD) of a host by a pathogenic Vibrio species, AphA is abundantly expressed to act as a master activator of genes responsible for virulence and biofilm formation, which promotes bacterial colonization and infection; when a HCD is reached, HMR is abundantly produced to act as a master repressor of virulence and biofilm formation.

### Cis-acting AphA Consensus

Two consensus constructs (a box sequence, and a PFM) were built herein to represent conserved cis-acting regulatory DNA signals recognized by the AphA orthologs from nine Vibrio species, V. parahaemolyticus, Vibrio sp. Ex25, Vibrio sp. EJY3, V. harveyi, V. vulnificus, V. splendidus, V. anguillarum, V. cholerae, and V. furnissii. The 20 bp AphA box sequence contains imperfect 7-nt inverted repeats with a 6-nt centered spacer, and this dyad symmetry structure is consistent with the fact that AphA monomers are unstable by themselves and constitute a dimer that sits on one face of the target cis-acting DNA to fit symmetrically into the DNA-binding site [43]. It should be noted that the 20 bp box herein is essentially an extended sequence of the 18 bp one previously characterized in V. harveyi [44] and V. cholerae [30].

Both box and PFM can be used to statistically predict the presence of AphA box-like elements within the target promoter-proximal DNA sequences, for instance by using the web-based Regulatory Sequence Analysis Tools [23]. For prediction with box, a concise calculation of number of mismatched nucleotides is employed to match the target DNA sequence against the box [23]. Compared to box, PFM gave a much more comprehensive description of uneven nucleotide composition in each position (i.e., some nucleotides occurred much more frequently than others); therefore, prediction with PFM will be more accurate, which generates a weight score for each target gene, and a higher score denotes the higher probability of AphA-promoter DNA association (Table 2). This assay raised several additional potential direct AphA targets especially including those responsible for virulence and biofilm formation in V. parahaemolyticus, and the validation of AphA-mediated regulation of these potential target genes are underway.

### Molecular Regulation of QS Regulator-encoding Genes

The transcriptional repression of aphA by AphA/HMR has been characterized in V. harveyi [7], [11], V. cholerae [30], [33], and V. parahaemolyticus ([15] and this study). The promoter-proximal DNA regions of aphA from the nine Vibrio species were aligned in Fig. 9a, in which shown were translation and transcription starts, –35 and –10 core promoter elements for σ^70^ recognition, Shine-Dalgarno (SD) sequences for ribosome recognition, and AphA/OpaR box-like sequences representing conserved signals for recognition by AphA/OpaR. For all the nine Vibrio species tested, the OpaR-box like sequences were about 18 bp upstream of the AphA box-like ones that overlapped the -35 core promoter regions, indicating that the mechanism of AphA/HMR-mediated repression of aphA was conserved in vibrios.

The AphA-mediated repression of HMR genes luxR and opaR has been established in V. harveyi [7] and V. parahaemolyticus (this study), respectively. In addition, the auto-repression of HMRs, OpaR, LuxR, and HapR, has been characterized in V. parahaemolyticus [15], V. harveyi [45] and V. cholerae [46], respectively. The sequence alignment (Fig. 9b) showed that the AphA box-like sequences overlapped the –10 core promoter regions while the OpaR box-like ones were downstream of the transcription start sites for HMR genes from all the above nine Vibrio species tested. The direct association between AphA/HMR and target promoters would block the entry of RNA polymerase to repress the transcription of corresponding target genes, and this mechanism appeared to be conserved in vibrios.

The AphA-mediated repression of qrr2-4 has been established in V. harveyi and V. cholerae [7], and also confirmed in V. parahaemolyticus in this study. The detected binding of AphA to the qrr4 promoter-proximal region indicated the direct regulation of qrr4 by AphA in V. harveyi [7] and V. parahaemolyticus (this study). In addition, the present study further validated that AphA repressed the qrr2-3 transcription in an indirect manner since AphA could not bound to the promoter-proximal regions of qrr2-3 (the association between qrr2-3 promoter regions and AphA were not tested in V. harveyi [7]). AphA box-like sequences were found upstream of the -24 core promoter regions of qrr4 from all five Vibrio species of Clade I (V. parahaemolyticus, Vibrio sp. Ex25, Vibrio sp. EJY3, V. harveyi, and V. vulnificus) rather than Clade II or III (Fig. 9c), indicating that AphA might directly repress the qrr4 transcription in only these five Vibrio species. AphA-mediated repression of qrr4 must occur by a highly unusual mechanism, since AphA binds a site located about one hundred nucleotides upstream of the core -24 and -12 promoter regions. In contrast, AphA box-like sequences could not be predicted for qrr2-3 from all the nine Vibrio species tested.

## Supporting Information

Figure S1 **Primer extension assay for validation of non-polar mutation.** The aphA null mutant ΔaphA was generated from the wild-type (WT) strain RIMD 2210633, and then the complemented mutant strain C-aphA was constructed. As determined by several distinct methods (see text), the qrr2 transcription was under the negative control of AphA. Herein, an oligonucleotide primer, which was complementary to the RNA transcript of qrr2, was employed to detect the primer extension product that represented the relative mRNA level of qrr2 in WT, ΔaphA, and C-ΔaphA. The primer extension products were analyzed with 8 M urea–6% acrylamide sequencing gel. Lanes C, T, A, and G represented Sanger sequencing reactions. The transcription start site of qrr2 was underlined in the DNA sequence. The qrr2 mRNA level was significantly enhanced in ΔaphA relative to WT, while no obvious change in the qrr2 transcription was observed between WT and C-aphA, which confirmed that the detecting enhanced transcription of qrr2 in ΔaphA was due to the aphA mutation rather than a polar mutation.
(TIF)
