# Supplementary figures and images for: Molecular Characterization of Direct Target Genes and cis-Acting Consensus Recognized by Quorum-Sensing Regulator AphA in Vibrio parahaemolyticus
Source: PLoS One. 2012 Sep 12;7(9):e44210. doi: 10.1371/journal.pone.0044210 (PMC3440409; doi:10.1371/journal.pone.0044210)

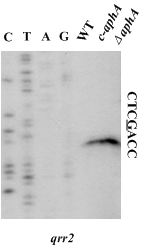

Supplement: Figure S1 — Primer extension assay for validation of non-polar mutation. The aphA null mutant ΔaphA was generated from the wild-type (WT) strain RIMD 2210633, and then the complemented mutant strain C-aphA was constructed. As determined by several distinct methods (see text), the qrr2 transcription was under the negative control of AphA. Herein, an oligonucleotide primer, which was complementary to the RNA transcript of qrr2, was employed to detect the primer extension product that represented the relative mRNA level of qrr2 in WT, ΔaphA, and C-ΔaphA. The primer extension products were analyzed with 8 M urea–6% acrylamide sequencing gel. Lanes C, T, A, and G represented Sanger sequencing reactions. The transcription start site of qrr2 was underlined in the DNA sequence. The qrr2 mRNA level was significantly enhanced in ΔaphA relative to WT, while no obvious change in the qrr2 transcription was observed between WT and C-aphA, which confirmed that the detecting enhanced transcription of qrr2 in ΔaphA was due to the aphA mutation rather than a polar mutation. (TIF) [file pone.0044210.s001.tif]
